# Supplementary material for: Study on Fluorescence Recognition of Fe3+, Cr2O72− and p-Nitrophenol by a Cadmium Complex and Related Mechanism
Source: Molecules. 2023 Feb 15;28(4):1848. doi: 10.3390/molecules28041848 (PMC9965397; doi:10.3390/molecules28041848)
Supplement: Supplementary file 1 [file molecules-28-01848-s001.zip › molecules-2149372-supplementary.pdf]

*Supplementary Materials for the Manuscript:*

# Study on Fluorescence Recognition of $Fe^{3+}$, $Cr_2O_7^{2-}$ and *p*-Nitrophenol by a Cadmium Complex and Related Mechanism

**Lu Liu [1], Jian-Min Li [2], Hui-Jie Wang [1], Meng-Di Zhang [1], Yu Xi [1], Jie Xu [1], Yuan-Yuan Huang [1], Bo Zhang [1], Ying Li [1], Zhen-Bei Zhang [1], Zi-Fang Zhao [1] and Cheng-Xing Cui [1,*]**

[1] School of Chemistry and Chemical Engineering, Henan Institute of Science and Technology, Xinxiang, 453003, China
[2] School of Resources and Environment, Henan Institute of Science and Technology, Xinxiang 453003, China
* Correspondence: chengxingcui@hist.edu.cn

**Table S1 Crystal Data and Structure Refinement Details for 1.**

| Complex | 1 |
|---|---|
| formula | $C_{82}H_{66}Cd_2N_{12}O_{13}S_4$ |
| fw | 1780.50 |
| *T*/K | 293(2) |
| $\lambda$ (Cu K$\alpha$), Å | 1.54184 |
| Cryst syst | triclinic |
| Space group | *P-1* |
| a (Å) | 12.8204(5) |
| b (Å) | 12.9684(5) |
| c (Å) | 13.2324(5) |
| $\alpha$(°) | 107.995(3) |
| β(°) | 95.395(3) |
| γ(°) | 110.667(4) |
| V (Å$^3$) | 1906.10(14) |
| *Z* | 1 |
| $D_{calcd}$.(g·cm$^{-3}$) | 1.551 |
| abs coeff/mm$^{-1}$ | 6.103 |
| *F*(000) | 906.0 |
| $2\theta$ (°) | 7.214–141.906 |
| GOF | 1.048 |
| $R_1$(I > 2sigma(I))[a] | 0.0497 |
| $wR_2$(I > 2sigma(I))[b] | 0.0584 |

[a]$R_1 = \sum||F_o|-|F_c||/\sum|F_o|$. [b]$wR_2 = [\sum w(F_o^2 - F_c^2)^2/\sum w(F_o^2)^2]^{1/2.}$

**Table S2.** Selected Bond Lengths (Å) and Bond Angles (deg) for 1[a.]

| Complex 1 | | | | | |
|---|---|---|---|---|---|
| **Cd(1)-N(1)** | **2.349(3)** | **Cd(1)-N(2)** | **2.378(3)** | **Cd(1)-N(5)$^1$** | 2.419(3) |
| Cd(1)-N(6)$^1$ | 2.391(4) | Cd(1)-O(1) | 2.325(3) | Cd(1)-O(6)$^1$ | 2.240(3) |
| Cd(1)$^1$-N(5) | 2.419(3) | Cd(1)$^1$-N(6) | 2.391(4) | Cd(1)$^2$-O(6) | 2.240(3) |
| N(1)-Cd(1)-N(2) | 69.96(12) | N(1)-Cd(1)-N(5)$^1$ | 98.73(12) | N(1)-Cd(1)-N(6)$^1$ | 161.48(12) |
| N(2)-Cd(1)-N(5)$^1$ | 81.43(12) | N(2)-Cd(1)-N(6)$^1$ | 95.13(12) | N(6)$^1$-Cd(1)-N(5)$^1$ | 67.32(12) |
| O(1)-Cd(1)-N(1) | 90.52(14) | O(1)-Cd(1)-N(2) | 150.63(14) | O(1)-Cd(1)-N(5)$^1$ | 80.12(12) |
| O(1)-Cd(1)-N(6)$^1$ | 98.56(14) | O(6)$^2$-Cd(1)-N(5)$^1$ | 142.44(13) | O(6)$^2$-Cd(1)-N(6)$^1$ | 81.37(13) |
| O(6)$^2$-Cd(1)-O(1) | 84.79(15) | | | | |

[a] Symmetry transformations used to generate equivalent atoms in complex (**1**): $^1$ 2−x,3−y,2−z; $^2$ 1−x,1−y,1−z.

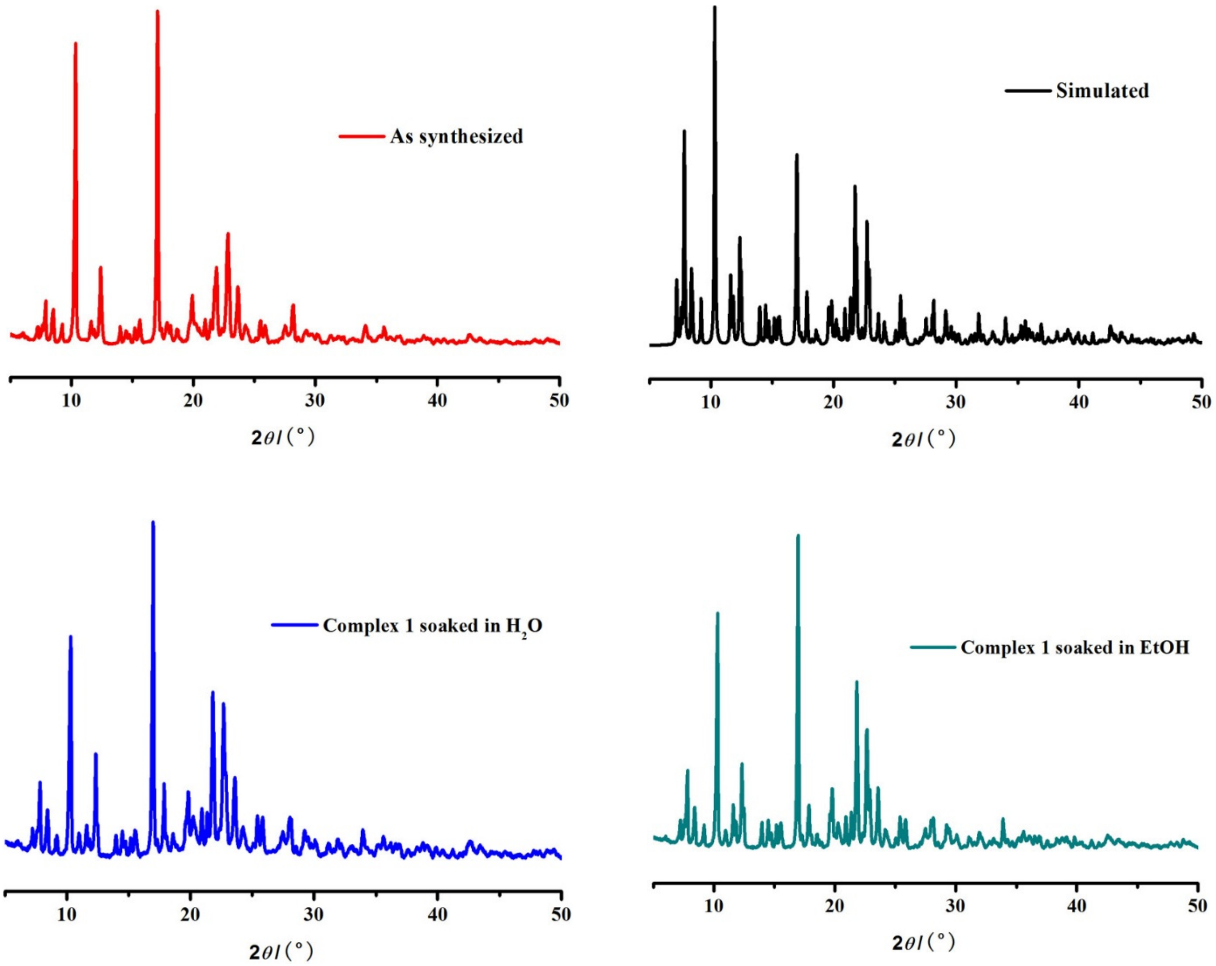


**Figure S1.** Experimental (red)、simulated (black)、soaked in $H_2O$ (blue) and soaked in EtOH (cyan) PXRD patterns of complex **1**.

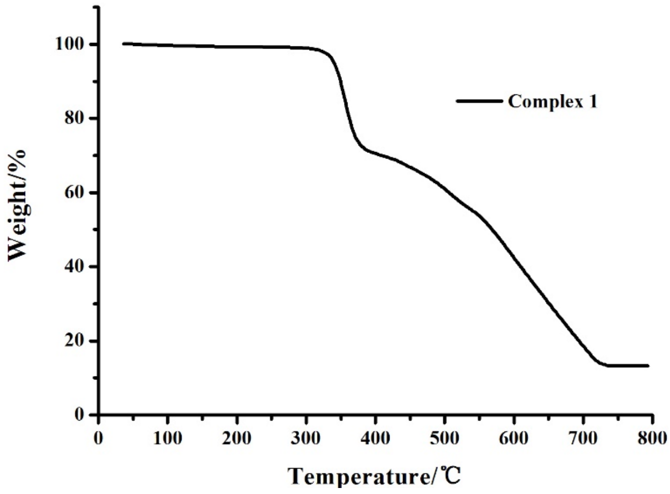

**Figure S2.** Thermogravimetric curve of complex **1**.

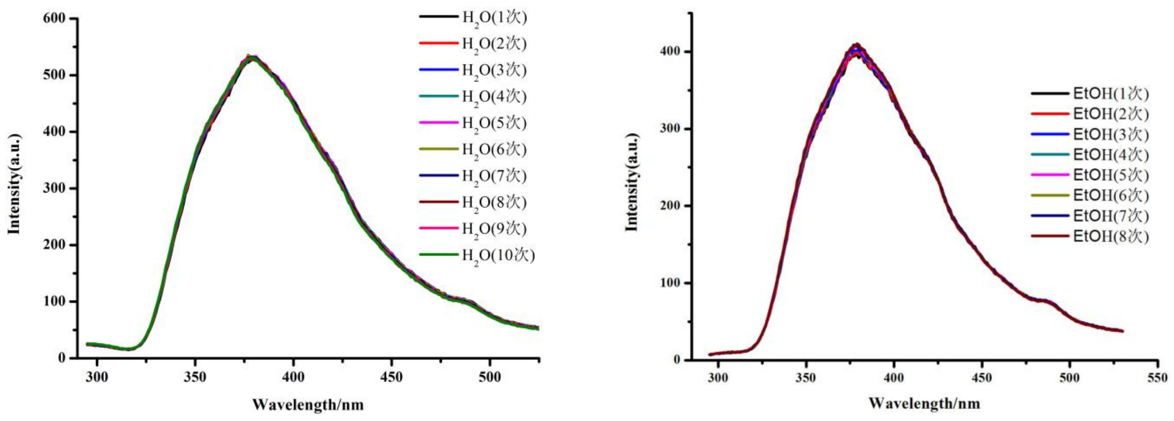


(**a**) (**b**)

**Figure S3.** Fluorescence spectra tested 10 times in water (**a**) and tested 8 times in EtOH (**b**) of complex **1.**

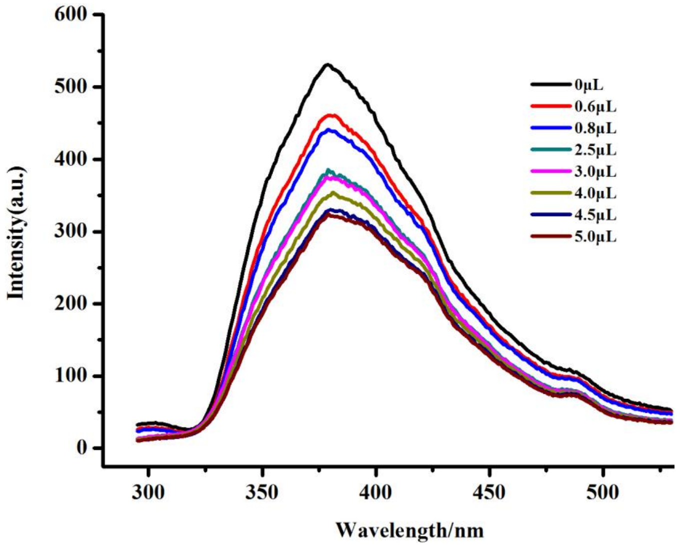


(**a**)

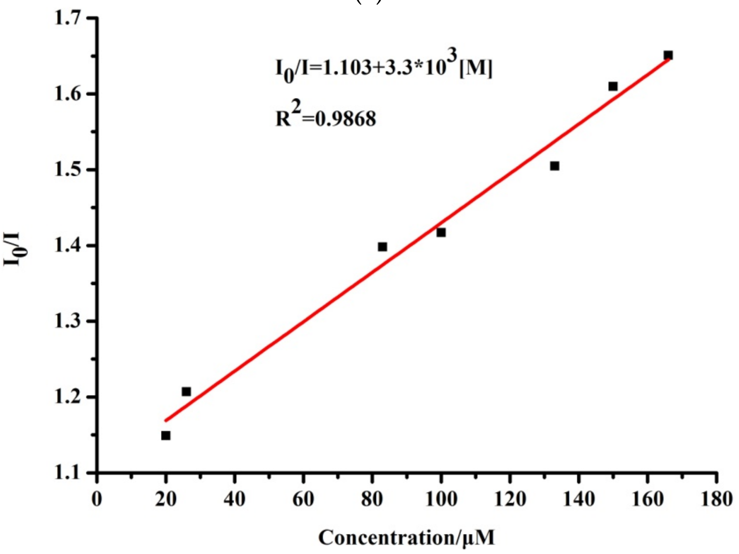


(**b**)

**Figure S4.** (**a**) Fluorescence spectra of complex **1** dispersed in aqueous suspension upon incremental addition of $Fe^{3+}$ ions. (**b**) The linear correlation for the plot of $I_0/I$ vs concentration of $Fe^{3+}$.

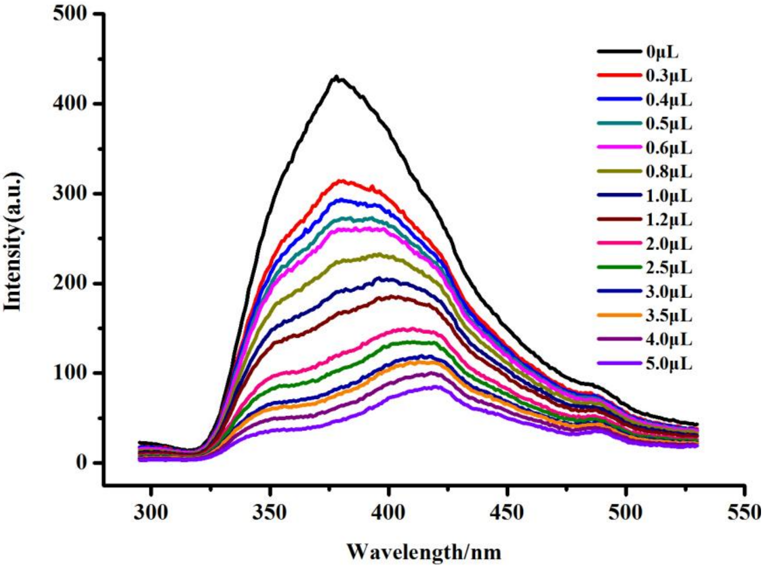


(**a**)

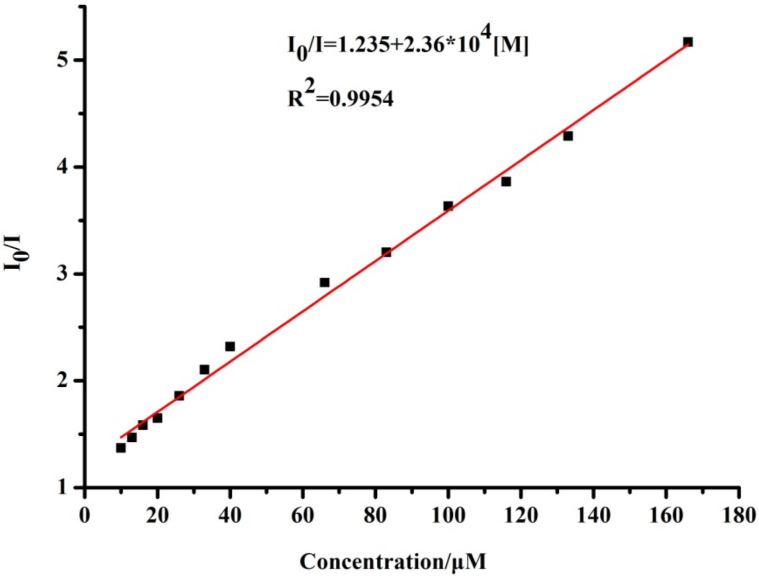


(**b**)

**Figure S5.** (**a**) Fluorescence spectra of complex **1** dispersed in aqueous suspension upon incremental addition of $Cr_2O_7^{2-}$ ions. (**b**) The linear correlation for the plot of $I_0/I$ vs concentration of $Cr_2O_7^{2-}$.

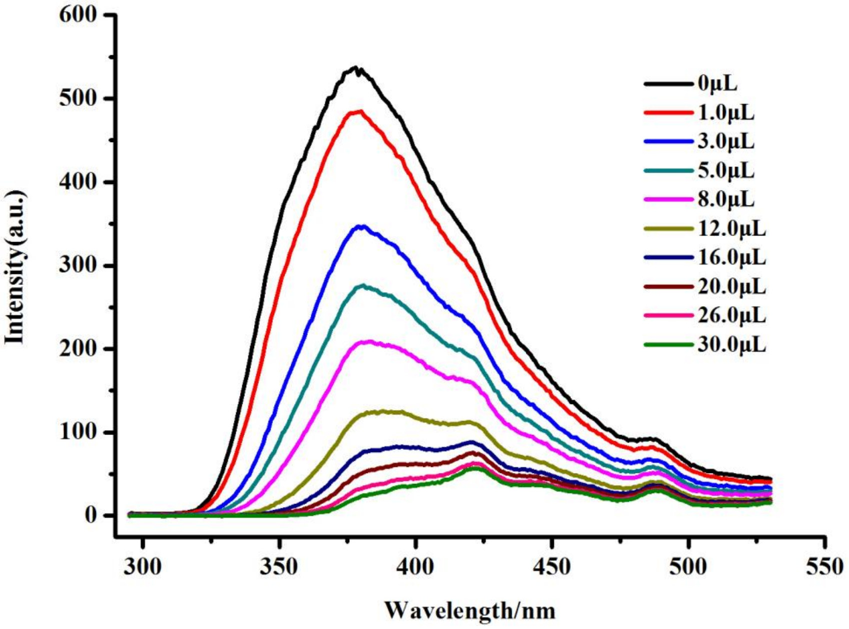


(**a**)

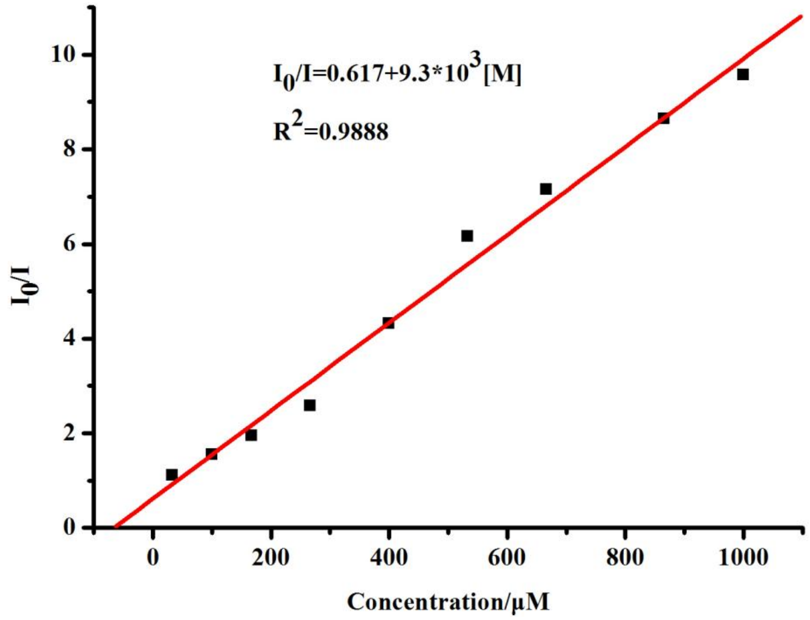


(**b**)

**Figure S6.** (**a**) Fluorescence spectra of complex **1** dispersed in aqueous suspension upon incremental addition of p-Nitrophenol. (**b**) The linear correlation for the plot of $I_0/I$ vs concentration of p-Nitrophenol.
